# THE IMPACT OF BREASTFEEDING ON THE NEONATAL ADAPTION OF INFANTS EXPOSED TO SSRIS AND SNRIS IN UTERO

**DOI:** 10.51894/001c.122898

**Published:** 2024-08-30

**Authors:** Torrey Halbert, Robin Blumer

**Affiliations:** 1 OBGYN Henry Ford Wyandotte

## 38

### INTRODUCTION

70 million individuals residing within the US are estimated to suffer from mental health disorders. Serotonin reuptake inhibitors (SSRI) and serotonin norepinephrine reuptake inhibitors (SNRI) are the most used medications for women with anxiety and depression disorders. With rising rates of anxiety and depression, the rate of use of SSRI and SNRIs during pregnancy is increasing. To date, research has not demonstrated significant teratogenic effects of these medications except for a slight increase in cardiac malformations. However there has been demonstrated impacts of SSRIs and SNRIs on the neonatal transition including neonatal abstinence syndrome (NAS) and increased rate of persistent pulmonary hypertension.

Neonatal abstinence syndrome (NAS) has been defined as disturbances in the central nervous, gastrointestinal, autonomic and respiratory systems. The Finnegan Neonatal Abstinence Scoring system was originally developed as an objective and periodic way to score infants exposed to opioids in utero. This scoring system has now been used to identify neonatal behavioral changes associated with multiple substances including SSRIs and SNRIs. Additionally, SSRIs and SNRIs have been found in breast milk, but no clinical effect of the neonate from the breast milk has been documented.

### OBJECTIVE

This study’s objective is to assess if breast milk fed infants have lower Finnegan NAS scores than fetus being fed with formula.

### METHODS

Data was obtained by querying the medical record of past patients who were using SSRIs or SNRIs at time of delivery in the Henry Ford Health Systems. Mothers using other substances that increase risk for NAS were excluded. Furthermore, the Finnegan NAS scores of the infants born to these mothers was extracted in a similar fashion. The scores between infants’ breast fed were compared to infants’ formula fed to assess for clinical significance.

### RESULTS

There was no clinical significance noted between infants’ breast fed as compared to infants’ formula fed in regard to their NAS score.

### CONCLUSION

This study will help offer information to expectant mothers to help make informed medical decisions for both them and their child.

